# Does Nutritional Supplementation Have a Disease-Modifying Effect on the Alzheimer’s Disease Neurodegenerative Process?

**DOI:** 10.14283/jarlife.2024.10

**Published:** 2024-05-22

**Authors:** K.V. Giudici

**Affiliations:** 1. Institute of Aging, Gerontopole of Toulouse, Toulouse University Hospital, Université Toulouse III Paul Sabatier, Toulouse, France.

**Keywords:** Alzheimer’s, supplementation, amyloid, tau, cognitive decline, aging

## Abstract

Because nutrition is one of the main factors related to Alzheimer’s disease (AD), questions arise about how taking nutrients as supplements can affect its pathophysiological process. In the present study, an overview of the potential effects of nutritional supplementation on the main biomarkers related to the AD pathophysiology (i.e., amyloid-β and tau) is explored. Trials testing the supplementation of single or combined nutrients versus placebo identified effects on some AD biomarkers, but changes were not always accompanied by positive effects on cognitive function. Differences in characteristics of studied populations (cognitive status, age, educational level), choice of nutrient combinations and doses, duration of intervention, and adjustments for potential confounders are some factors that may explain discrepancies in findings.

**Abbreviations**
Aβ: amyloid-β
AD: Alzheimer’s disease
ADAS-cog: Alzheimer disease assessment scale-cognitive subscale
ALA: α-lipoic acid
APOE: apolipoprotein E
CSF: cerebrospinal fluid
DHA: docosahexaenoic acid
EPA: eicosapentaenoic acid
IQ: intelligence quotient
MAPT: Multidomain Alzheimer Preventive Trial
MCI: mild cognitive impairment
MMSE: Mini Mental State Examination
PET: positron emission tomography
p-tau181: phosphorylated tau at threonine 181
PUFA: polyunsaturated fatty acids
RCT: randomized controlled trial
t-tau: total tau
VISP: Vitamin Intervention for Stroke Prevention

## Introduction

**T**he pathophysiological process leading to the characterization of Alzheimer’s disease (AD) as a unique neurodegenerative disorder, among other types of dementia, consists of the accumulation of amyloid-β (Aβ) plaques and pathologic tau deposits in the brain (1). The neurodegeneration influenced by these processes, coupled with dementia, results in gradual cognitive decline that may reach advanced stages in which quality of life is severely affected (2).

Although there is a strong genetic risk factor for the development of AD (the presence of the APOE ε4 allele) (3), many other factors such as diet, physical activity level, stress management and sleep quality are known to affect the probability of accumulating Aβ and contributing to tau phosphorylation and aggregation in the brain, and consequently increasing the risk of AD (4).

The detection and diagnosis of AD has been classically based on the evaluation of Aβ and tau biomarkers in the brain (by positron emission tomography – PET) or in the cerebrospinal fluid (CSF) (1, 2), which are either expensive or invasive methods. More recently, blood-based biomarkers have emerged as less complex alternatives, but with compatible reliability (5, 6). These measures have been used as outcomes in trials testing what can be done, in terms of lifestyle changes, to prevent or fight this neurodegenerative disease. Since nutrition is one of the main factors related to AD (7), questions arise if taking nutrients as supplements is able to affect its pathophysiological process. In the present study, an overview on the potential effects of nutritional supplementation on the main biomarkers related to the AD pathophysiology (i.e., Aβ and tau) is explored.

## Effects of nutritional supplementation on Aβ and tau biomarkers

The main omega-3 polyunsaturated fatty acids (PUFA) (eicosapentaenoic acid – EPA and docosahexaenoic acid – DHA) are known by their substantial anti-inflammatory and antioxidant properties (8). DHA is especially important to brain function: besides its essential structural properties, it regulates synaptic function, modulates gene expression, acts as an indirect antioxidant and contributes to neuroprotection (9). The Multidomain Alzheimer Preventive Trial (MAPT) explored the effects of a 3-year supplementation with omega-3 PUFA (800mg DHA and 225mg EPA/day), alone or combined to physical activity and cognitive training, on AD biomarkers and clinical tests among 1,680 community-dwelling older adults living in France and Monaco (10, 11). At the end of the 3-year follow-up, no effects of interventions were observed in cognitive function (evaluated with a composite cognitive score) (11), nor in plasma phosphorylated tau at threonine 181 (p-tau181) when a subsample of 527 participants with this measure was analyzed (12). Interestingly, in another secondary analysis of MAPT among a subsample of 483 participants with plasma Aβ42/40 ratio assessments, the combined intervention showed a positive effect on cognitive function in the per-protocol positive amyloid group (i.e., Aβ42/40≤0.0107; n=154), after 1 year and 3 years. However, no differences were found between intervention and placebo groups after two additional years of observational follow-up (13).

In the OmegAD Study, a 6-month omega-3 PUFA supplementation (2.3g/day) or placebo was offered to 35 patients diagnosed with AD. Compared to placebo, intervention did not affect CSF Aβ38, Aβ40, Aβ42, total tau (t-tau) and p-tau (14, 15). A secondary analysis with 33 participants revealed that changes in CSF levels of DHA due to supplementation were inversely correlated with CSF levels of t-tau and p-tau, indicating that the more DHA increased in CSF, greater was the change in CSF tau biomarkers (16). Another trial offered 2g/day of DHA or placebo for 240 individuals with mild cognitive impairment (MCI) living in China over 2 years, and found decreases in blood Aβ42 levels and expression of Aβ protein precursor mRNA, which were accompanied by increases in scores of full-scale intelligence quotient (IQ), verbal IQ and subdomains of information and digit span, among those taking the DHA supplement (17).

Vitamin D is another nutrient believed to contribute to the development of cognition and its maintenance over time (18). Acting as a hormone with multiple actions in metabolism, it impacts neurocognition by inducing neuroprotection, modulating oxidative stress, regulating calcium homeostasis and inhibition inflammation (19). A trial with 210 participants testing a 1-year vitamin D supplementation (800IU/day) in older adults with AD observed a decrease in plasma Aβ42 and improvements in cognitive assessments (information, arithmetic, digit span, vocabulary, block design and picture arrange scores) among the intervention group (20). Another trial testing the effect of a high-dose short-term vitamin D supplementation (50,000IU/week for 8 weeks) versus placebo on plasma Aβ40 of 24 vitamin D insufficient adults observed a greater plasma Aβ40 increase among the intervention group, what authors suggested to be an indicative of decreased brain Aβ (21).

Excessive homocysteine (Hcy) has direct neurotoxic effects, due to inducing oxidative stress, causing DNA damage and apoptosis, and stimulating Aβ deposition in the brain (22). Some B-vitamins are known by their role in Hcy remethylation, thus contributing to decrease Hcy levels and to potentially avoid its neurotoxicity (23). Chen et al. (24) explored the effects of a 6-month folate supplementation (1.25mg/day) on inflammatory biomarkers and cognitive function among patients with AD. They found that plasma Aβ40 levels were lower, Aβ42/40 ratio was higher and mean Mini Mental State Examination (MMSE) score was slightly increased in the intervention group compared to the control group at the end of follow-up. In the Vitamin Intervention for Stroke Prevention (VISP) trial, 300 adults with ischemic stroke and high total Hcy (tHcy) levels (a risk factor for AD) were treated with either a high-dose supplement (composed of 25mg of pyridoxine, 0.4mg of cobalamin, and 2.5mg of folic acid) or a low-dose supplement (200mcg of pyridoxine, 6mcg of cobalamin, and 20mcg of folic acid) for 2 years (25). At the end of follow-up, no alterations were observed for Aβ40, Aβ42 or Aβ42/40 ratio. On the other hand, tHcy levels significantly decreased in both groups (more among participants taking the high dose) and were strongly correlated with plasma Aβ40, but not with Aβ42 concentrations (25).

Important copper concentrations and localization changes have been identified in AD cerebral regions, raising questions whether therapeutic approaches for regulating its levels could affect AD pathophysiology (26). Kessler et al. (27) offered a 12-month supplementation of 8mg/day of this nutrient or placebo to a sample of patients with mild AD. They found no effect on the progression of CSF tau and p-tau levels nor in MMSE and Alzheimer disease assessment scale-cognitive subscale (ADAS-cog) scores. Still, a lower decline in CSF Aβ42 was observed in the intervention group (a positive finding, since decreased CSF Aβ42 is a diagnostic marker for AD).

The Nolan Study, in turn, tested the effect of a 1-year multi-nutrient supplementation (including omega-3 PUFA, vitamin C, vitamin D, vitamin E, thiamin, riboflavin, niacin, pantothenic acid, pyridoxine, folic acid, biotin, cobalamin, selenium, choline and citrulline) on clinical tests, imaging and blood biomarkers related to the AD pathology among a sample of 362 community-dwelling older adults living in France (28, 29). At the end of the follow-up, supplementation could not postpone the increase in plasma p-tau181 (observed in both intervention and placebo groups) (29), and neither showed an effect on cognitive function (28). Another randomized controlled trial (RCT) found no benefits of a 16-week supplementation of combined vitamin E (800IU/day), vitamin C (500mg/day) and α-lipoic acid (ALA) (900mg/day), or coenzyme Q alone (1200mg/day) on CSF Aβ42, t-tau or p-tau181 in a sample of 66 subjects with mild to moderate AD (30). Surprisingly, a faster decline in MMSE score was identified among the group receiving vitamin E, vitamin C and ALA (30).

## Conclusions and perspectives

Despite the well-established influence of diet in the development of the AD neurodegenerative process (7), it is still not clear how nutritional supplementation may contribute to preventing or postponing it and, consequently, to protect cognitive function. Trials currently show that some biomarkers related to the AD development can be modified with supplementation protocols varying from months to years. Still, changes are not always accompanied by positive effects on cognitive function. Differences in characteristics of studied populations (cognitively normal participants or subjects with MCI or AD, age ranges, educational level), choice of nutrients’ combinations and doses, duration of interventions and adjustments for potential confounders (such as APOE ε4 status) are some factors that may explain discrepancies in findings.

It is known that AD slowly develops for decades before cognitive decline is perceptible and starts negatively affecting a person’s life (2). It is thus comprehensible that nutritional supplementation alone in advanced age seem to be not able to neutralize the decades of metabolic processes that have been slowly acting on a person’s body and brain (and triggered not only by inadequate diet, but also by other lifestyle factors as stress, bad sleep quality and sedentary behavior (31), leading to the development of AD.

In spite of increasing costs and complexity, future research on the topic might benefit of enhanced sample sizes and/or duration of interventions (covering a higher percentage of average lifespan) in study protocols. Another point to consider is that not every person may benefit from supplementation. In this sense, scanning for nutritional deficiencies related to cognitive performance may help identify individuals for whom supplementation would be more probably effective. Moreover, genetic variants are able to affect the way nutrients act on metabolism. In AD, the APOE ε4 allele is recognized as the major genetic risk factor in late onset Alzheimer’s (3), partly due to impairing lipid transport from neurons to astrocytes (32), altering microglia function (33), impairing neuronal insulin signaling (34), favoring blood-brain barrier dysfunction (35) and increasing DHA β-oxidation (36). Thus, studies taking into account major polymorphisms related to AD physiopathology take a step forward in this investigation.

Current and growing knowledge on the theme must be used to support a careful choice of nutrients in future studies. Since evidence points towards oxidative stress as an early event leading to Aβ deposition and dimerization of tau protein and its subsequent hyperphosphorylation (37, 38), therapeutic approaches focusing on antioxidants (as vitamin C, vitamin E and selenium) might be considered. Additionally, inflammation (39) and impaired glucose metabolism (40) are both related to the development of AD, and might be the target of nutritional interventions as well – by offering, for example, nutrients with anti-inflammatory abilities (as omega-3 PUFA (8) and folate (24)), and nutrients known to improve glucose homeostasis (as vitamin D (41)).

Finally, it should be kept in mind that nutritional supplementation does not aim to overlap the importance of following balanced dietary patterns over the life course in order to prevent AD and other diseases – even because, to date, nutrients synergy as experienced with food intake cannot be replicated with supplements. Notwithstanding, identifying specific nutrients or bioactive compounds for which a high dose (incompatible with usual food intake) would be effective in fighting the AD pathophysiological process is another factor that may justify supplementation. Altogether, the state of art points towards this as a hot topic in research, for which further important discoveries are yet to be achieved.
